# Supplementary material for: Rychc Confers Extreme Resistance to Potato virus Y in Potato
Source: Cells. 2022 Aug 18;11(16):2577. doi: 10.3390/cells11162577 (PMC9406545; doi:10.3390/cells11162577)
Supplement: Supplementary file 1 [file cells-11-02577-s001.zip › cells-1846636-supplementary.pdf]

**Supplemental Tables**

**Table S1.** Genetic markers used for genetic mapping

| Primer name | Forward primer (5'->3') | Tm (℃) | Comment product size(bp) |
|---|---|---|---|
| M4-F | ACCAGCAGACAGTTGTTATG | 56 | DNA marker |
| M4-R | AACTCAGTGTGCGGATTC | | |
| M2425-2F | TCTTCATCTCTTCCTAAGTG | 55 | DNA marker |
| M2425-2R | AACCTCCTTGTGATGTATT | | |
| M2526-F | AACTAGAAGCCAGAATCTAACG | 56 | DNA marker |
| M2526-R | GGTCTTACCAACAAGTCATACC | | |
| M28-F | TTGGCATCATCATCTTAGCA | 56 | DNA marker |
| M28-R | ACCATCAATGTCCCAGTTC | | |
| M71-20-F | GTAACCATTGTCTAA-GCCGAAAG | 55 | DNA marker |
| M71-20-R | TAAGCTACCAGAACCAAAC-CAAC | | |
| M71-21-1F | CCCTCGGTTTGTATTATTCCTTC | 55 | DNA marker |
| M71-21-1R | TCCGCCATGTGTGTATTTGTGAC | | |
| M71-28-3F | GGCTGCTCGATCACAATCACAAC | 55 | DNA marker |
| M71-28-3R | CACGAC-CAAACTGAAATCCCCAC | | |
| M64-16-F | ATGGCAAAGTTAA-GAGGTATATCAG | 55 | DNA marker |
| M64-16-R | TTATGTGGCACAC-TCTGTTACTCC | | |
| MG64-15-F | AAACACACCTTGAAATTAG-TAAGC | 55 | DNA marker |
| MG64-15-R | GAAGGTTTATGGTGGTTTCCGCG | | |
| MG64-17-F | TAAGTAAGAAAC-CTACTTATTCTCCG | 55 | DNA marker |
| MG64-17-R | GTTTGACAACCTCCCTAG-TATAAA | | |
| MG64-17-1F | ACAACGTCTCTGCCAGTGCGATC | 55 | DNA marker |
| MG64-17-1R | ACCGTTGTTGAGCTACTTGTT | | |
| MG64-17-2F | TTATTCCTTGTCCCAATCGACAC | 55 | DNA marker |
| MG64-17-2R | TGGGATAAACATGCAACGCAC | | |
| M64-17-F | CCATCTTGAGTTTGTATTGTG-CATT | 55 | DNA marker |

| | | | |
|---|---|---|---|
| M64-17-R | AGCACCAGCACTAATGAAA-TAAAGG | | |
| SNP48-F | ACTGTCAACCTCCAAAAAC-CAAA | 56 | SNP marker |
| SNP48-R | GGGGTGGGGGATTCAGACTT | | |
| M4950-1F | TAACTGATGCGATCTAATGA-TAGGG | 58 | DNA marker |
| M4950-1R | GGTAATGCGAGAATTTCCACAA | | |
| M4950-2F | GATAAGGTAAGGCTTTGTGTACG | 58 | DNA marker |
| M4950-2R | GAGTTTTAGTTGATGGCGGATA | | |
| M50-F | GCAAGAAAGTACCACCCAGAT | 58 | DNA marker |
| M50-R | AGAAGTCAAGGGATTCAACGT | | |
| M32-F | ATCGCTTCAAAAATCACCA | 51 | DNA marker |
| M32-R | AAAGGCTTTAACAACTCCG | | |
| M152-F | TTGATTGGAGTTAAGGGTGGT | 54 | DNA marker |
| M152-R | CGTGAGGTCTATAAGGAGGGT | | |
| M236-F | AGCCAAAGCAAGTTCAAAT | 52 | DNA marker |
| M236-R | ACAGAAGCCACAACACAAA | | |

**Table S2.** Primers used in this study

| Primer name | Forward primer (5'->3') |
|---|---|
| pBI-OAC-C3-F | AGAACACGGGGGACTCTAGAATGAATACTCAAGGA-GAATCATCAACTACT |
| pBI-OAC-C3-R | GATCGGGGAAATTCGAGCTCTTATGCCTTCCGGA-TACTACTCTGCTG |
| pBI-OAC-C4-F | AGAACACGGGGGACTCTAGAATGAATACTCAAGGA-GAATCATCTTC |
| pBI-OAC-C4-R | GATCGGGGAAATTCGAGCTCTTATTCTCCGGA-GAAACATTTTCC |
| pBI-OE3-C4-pro-moter-F | ACCATGATTACGCCAAGCTTGTCGACATGAA-TACTCAAGGAGAATCATCTTC |
| pBI-OE3-C4-pro-moter-R | GATCGGGGAAATTCGAGCTCCTGA-TATACAGGCGAATTCAGAAC |
| pBI-OE3-C4-gene-F | ATTACGCCAAGCTTGTCGACGGCAAAAGTTAGATTAC-GAAAATACC |
| pBI-OE3-C4-gene-R | CTCCTTGAGTATTCATGTCGACATTTGATG |
| qPCR-C4-F | GAAGATACTCGCAAAAACTTCATTGA |
| qPCR-C4-R | TTCGAGAAAACAACAATGGCAATT |
| qPCR-PVY-CP-F | ACACCAGTGAGGGCTAGRGA |
| qPCR-PVY-CP-R | GTGGTGTGCCTCTCTGTGTT |
| Semi-qPCR-C3-F | GCGAACCTGAGCACCAAGAC |
| Semi-qPCR-C3-R | TCCGCAGGAAAATGTGATGGTA |
| Semi-qPCR-C4-F (MG64-17-1F) | ACAACGTCTCTGCCAGTGCGATC |
| Semi-qPCR-C4-R (MG64-17-1R) | ACCGTTGTTGAGCTACTTGTT |
| Ef1$\alpha$-F | ATTGGAAACGGATATGCTCCA |
| Ef1$\alpha$-R | TCCTTACCTGAACGCCTGTCA |

**Table S3.** Statistical results from the potato BC-SNP map

| Chromosome | Length of linkage group (cM) | Interval numbers | No. of markers | Mean marker intervals (cM) | Max Marker interval (cM) |
|---|---|---|---|---|---|
| 1 | 129.92 | 17 | 22 | 5.91 | 29.62 |
| 2 | 81.25 | 23 | 27 | 3.01 | 14.67 |
| 3 | 79.80 | 20 | 31 | 2.57 | 18.97 |
| 4 | 86.14 | 11 | 21 | 4.10 | 27.96 |
| 5 | 85.33 | 10 | 17 | 5.02 | 17.1 |
| 6 | 67.97 | 8 | 11 | 6.18 | 16.74 |
| 7 | 69.65 | 15 | 31 | 2.25 | 13.83 |
| 8 | 65.43 | 14 | 18 | 3.63 | 16.76 |
| 9 | 52.80 | 6 | 9 | 5.87 | 31.97 |
| 10 | 68.08 | 9 | 11 | 6.19 | 27.94 |
| 11 | 68.14 | 15 | 23 | 2.96 | 15.14 |
| 12 | 64.31 | 12 | 16 | 4.02 | 13.52 |
| Total | 918.82 | 13 | 237 | | |
| Average | 76.57 | 160 | 19.75 | 6.3 | |

**Table S4.** Position and sequence of 6 tightly linked SNPs markers

| Name | Superscaffold | Size | Position | Sequence |
|---|---|---|---|---|
| solcap_snp_c2_22070 | PGSC0003DMB000000439 | 101 | chromosome 9 46389432-46389532 | AATCTACATCATCTGAG-TGAATTTCTAGCTGGTAA-GCGCGCTGATGGTCC[A/C]AAA-GAAGCTCTTCAAATTCTG-GACATTGTTTTGAGA-GAGCTTTCGATCAA |
| solcap_snp_c2_20640 | PGSC0003DMB000000020 | 101 | chromosome 9 48863726-48863826 | CGGACCCATT-GTCGCGCTGACCCACCCGAT-TATAACGAGAATCTG-GATTA[C/T]ATGCGCCCCGAG-TAG-TTTCCCTCCTCCACCCATGAA-TAACCCGTGAGGTC |
| solcap_snp_c2_40086 | PGSC0003DMB000000020 | 101 | chromosome 9 49152274-49152374 | GAACAGATCCTTCAA-GCATCCTCATTGAATATCAAC-GATCAATCAGGTGA[T/C]AGA-GAGATGTGCGCGTTTACATATTT-GAGGATGAATCCTGAC-CTATTCCA |
| solcap_snp_c2_40079 | PGSC0003DMB000000690 | 101 | chromosome 9 49247381-49247481 | GACGTAGTTGTCAT-ACCAGCTGGCGTTCCAAGAAA-GCCTGGTATGACACG[G/A]GAC-GACTTGTTCAACATTAATGCTAA-TATTGTGAAAGGCTTGGTT-GAGGC |
| solcap_snp_c2_40085 | PGSC0003DMB000000020 | 101 | chromosome 9 49152340-49152440 | TTGTTT-GCGTCTTTTCCTTCTTTCATTTTCTTCACGAAGGCAAC-GAATCG[T/C]CTCCAGTTATCAG-GATGGAATAGGTCAGGAT-TCATCCTCAAATATGTAAA |
| solcap_snp_c2_48042 | PGSC0003DMB000000339 | 101 | chromosome 9 51381163-51381263 | CTGAGGAA-TATACTCATTATTTTT-GCCAAACTCAATCCTGGTATAA-GATA[C/T]GTG-CAAGGGATGAATGAAGTATTGGCTCCACTATTCTATGTATTCAG-GAA |

**Table S5.** ELISA results for 5 recombinants

| Clones | ODA405 | | | | Average | Clones |
|---|---|---|---|---|---|---|
| | Rep1 | Rep 2 | Rep 3 | Rep 4 | | |
| 40-3 | 0.061 | 0.046 | 0.103 | 0.020 | 0.058±0.035 | R |
| 143-6 | 3.246 | 3.169 | 3.269 | 3.170 | 3.214±0.051 | S |
| Re1 | 3.490 | 3.520 | 3.543 | 3.245 | 3.449±0.138 | S |
| Re2 | 4.000 | 3.434 | 3.234 | 3.228 | 3.474±0.363 | S |
| Re3 | 0.045 | 0.043 | 0.036 | 0.041 | 0.041±0.004 | R |
| Re4 | 0.112 | 0.035 | 0.011 | 0.119 | 0.069±0.054 | R |
| Re5 | 0.089 | 0.171 | 0.012 | 0.010 | 0.071±0.076 | R |

*40-3 indicates *S. chacoense* accession 40-3, the resistant parent; 143-6 indicates *S. berthaultii* accession143-6, the susceptible parent.

**Table S6.** Functional annotation of 6 genes in the interval defined by fine mapping

| Gene | Conserved domain | Length (bp) |
|---|---|---|
| *C1* | No conserved domain | 5638 |
| *C2* | Oxidoreductase | 2198 |
| *C3* | TIR-NBS-LRR | 3940 |
| *C4* | TIR-NBS-LRR | 4167 |
| *C5* | No conserved domain | 549 |
| *C6* | No conserved domain | 3359 |

**Table S7.** Distribution of *$Ry_{chc}$* homologs in 13 wild accessions of *S. chacoense*

| | Copy number | No. of clones sequenced |
|---|---|---|
| *S. chacoense* accession40-3 | 15 | 57 |
| CHC39-7 | 20 | 75 |
| C545 | 8 | 74 |
| M6 | 5 | 24 |
| CHC524-8 | 6 | 41 |
| CHC524-8-1 | 4 | 24 |
| CHC524-8-2 | 6 | 48 |
| CHC54-1 | 20 | 71 |
| CHC54-2 | 18 | 53 |
| CHC54-3 | 8 | 36 |
| CHC55-1 | 20 | 78 |
| CHC55-2 | 10 | 60 |
| CHC55-3 | 20 | 63 |
| SUM | 160 | 704 |

**Table S8.** Genotyping of $Ry_{chc}$ in potato accessions with 3 molecular markers

| Name | Genotype | | | Phenotype |
|---|---|---|---|---|
| | M71-20 | MG64-17 | M64-17 | |
| *S. chacoense* accession40-3 | + | + | + | R |
| *S. berthaultii* accession143-6 | – | – | – | S |
| C545 | + | + | + | R |
| M6 | + | + | + | R |
| CHC39-7 | + | + | – | R |
| CHC524-8-1 | + | + | + | R |
| CHC524-8-2 | + | + | + | R |
| CHC524-8-3 | + | + | + | R |
| CHC54-1 | – | – | – | S |
| CHC54-2 | – | – | – | S |
| CHC54-3 | – | – | – | S |
| CHC55-1 | + | – | – | S |
| CHC55-2 | + | – | – | S |
| CHC55-3 | + | – | – | S |
| Eesteling | + | – | – | S |
| 03P74-16 | + | – | – | S |
| 06P13-6 | + | – | – | S |
| 07HE077-3 | + | – | – | S |
| 11FF28-9 | – | – | – | S |
| 392625-1 | + | – | – | S |
| 393140-2 | + | – | – | S |
| Agata | + | – | – | S |
| Da | – | – | – | S |
| F70021-2 | + | – | – | S |
| HB0211-1 | + | – | – | S |
| Monalisa | + | – | – | S |
| MPI 63.6131.63 | + | – | – | S |
| NEA20008 | + | – | – | S |
| Pepo418 | + | – | – | S |
| Riviera | – | – | – | S |
| S.punae | + | – | – | S |
| T0035-1 | + | – | – | S |

| | | | | |
|---|---|---|---|---|
| W2 | + | – | – | S |
| Дельфин | + | – | – | S |
| E10 | – | – | – | S |
| E3 | – | – | – | S |
| AC142 | + | – | – | S |

* –/+, absence/presence of the indicated molecular marker.

## Supplemental Figures

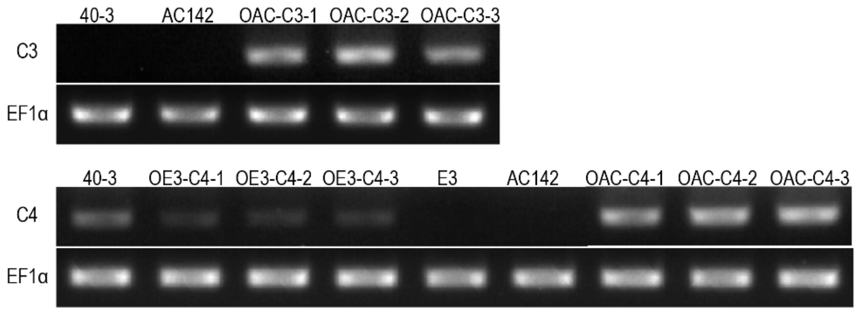


**Figure S1.** Expression analysis of *C3* and *C4* using semi-quantitative RT-PCR.

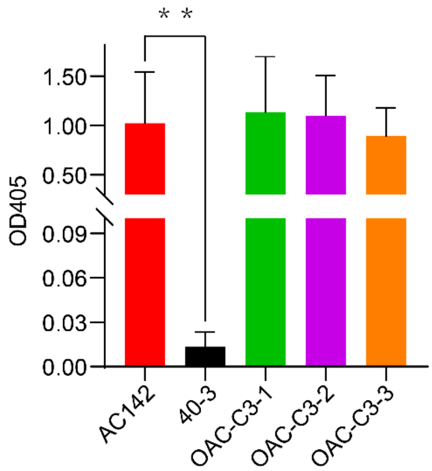


**Figure S2.** Phenotyping of PVY$^{O}$ resistance in *C3* transgenic lines. The phenotypic characterizations were performed with ELISAs. 40-3 indicates the resistant parent, *S. chacoense* accession 40-3. ELISA (ODA405) <0.1 indicates that the plants were resistant. ELISA (ODA405) >0.1 indicates that the plants were susceptible. Data are presented as mean values ± SE. ** indicates a statistically significant difference (P < 0.01) relative to AC142, as determined with a Student's t-test.

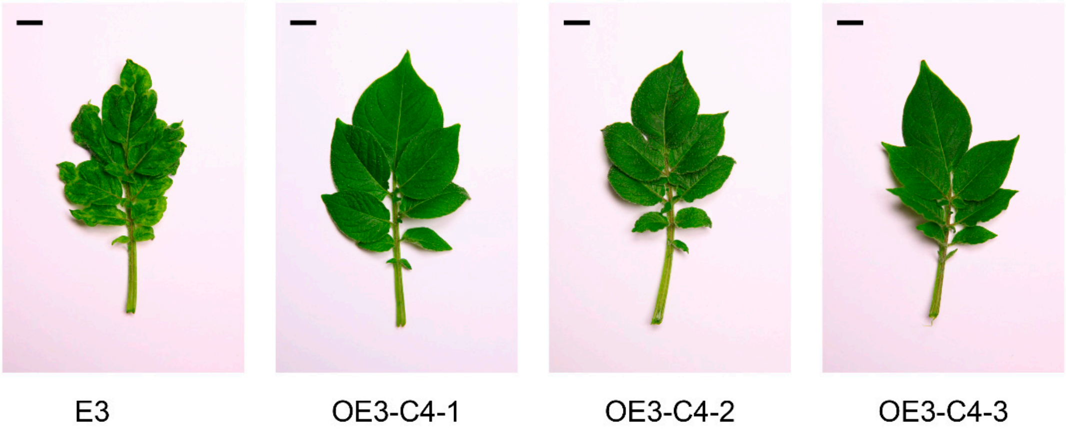


**Figure S3.** Phenotype of non-inoculated leaves of E3 transgenic strains approximately 1 month after virus inoculation.

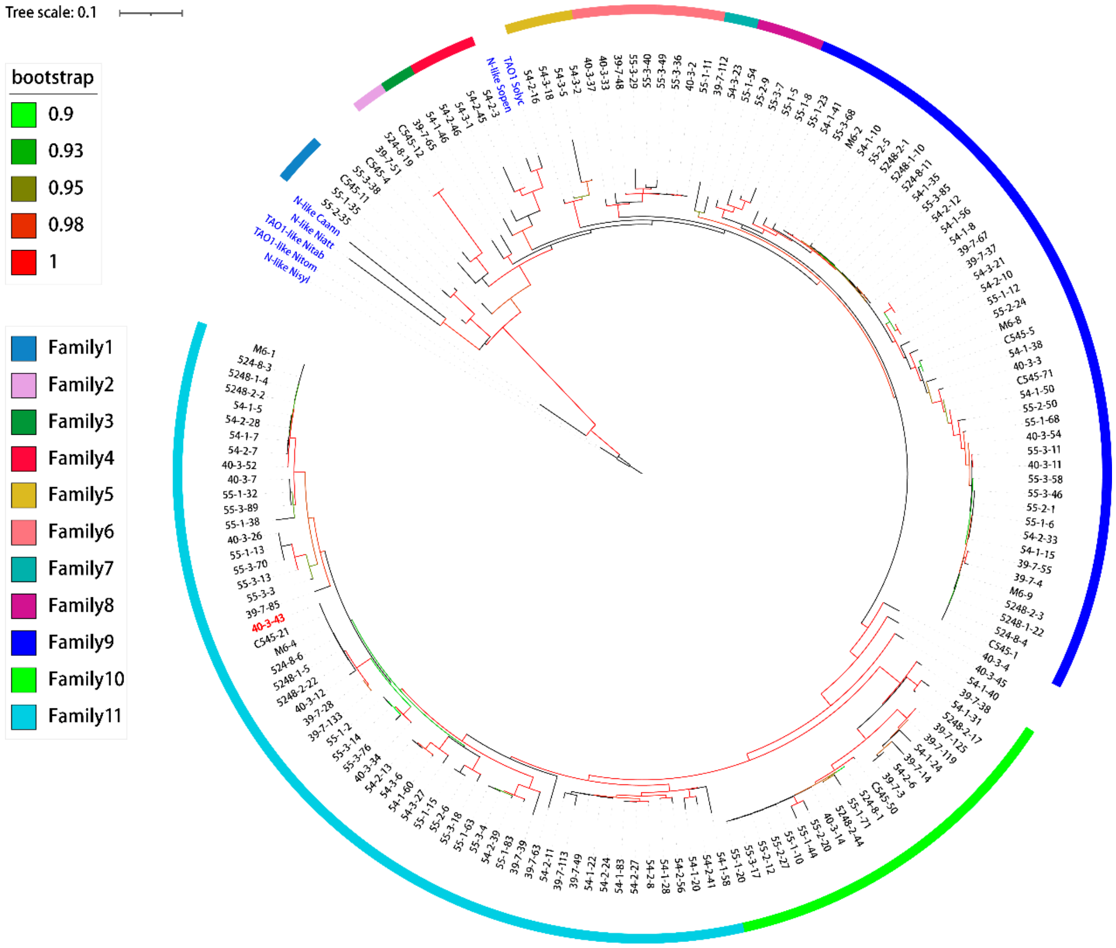


**Figure S4.** Phylogeny of *Ry*<sub></sub>*chc* homologs. A maximum likelihood (ML) phylogenetic tree was constructed for 13 diverse wild accessions of *S. chacoense*. Seven $Ry_{chc}$ homologs from other species (indicated in red) were used as outgroups. These seven homologs are *N-like_Caann* from pepper, *TAO1_Solyc* and *N-like_Sopen* from tomato, and *N-like_Niatt*, *N-like_Nisyl*, *TAO1-like_Nitab* and *TAO1-like_Nitom* from tobacco. The fragment of $Ry_{chc}$ is indicated with red.

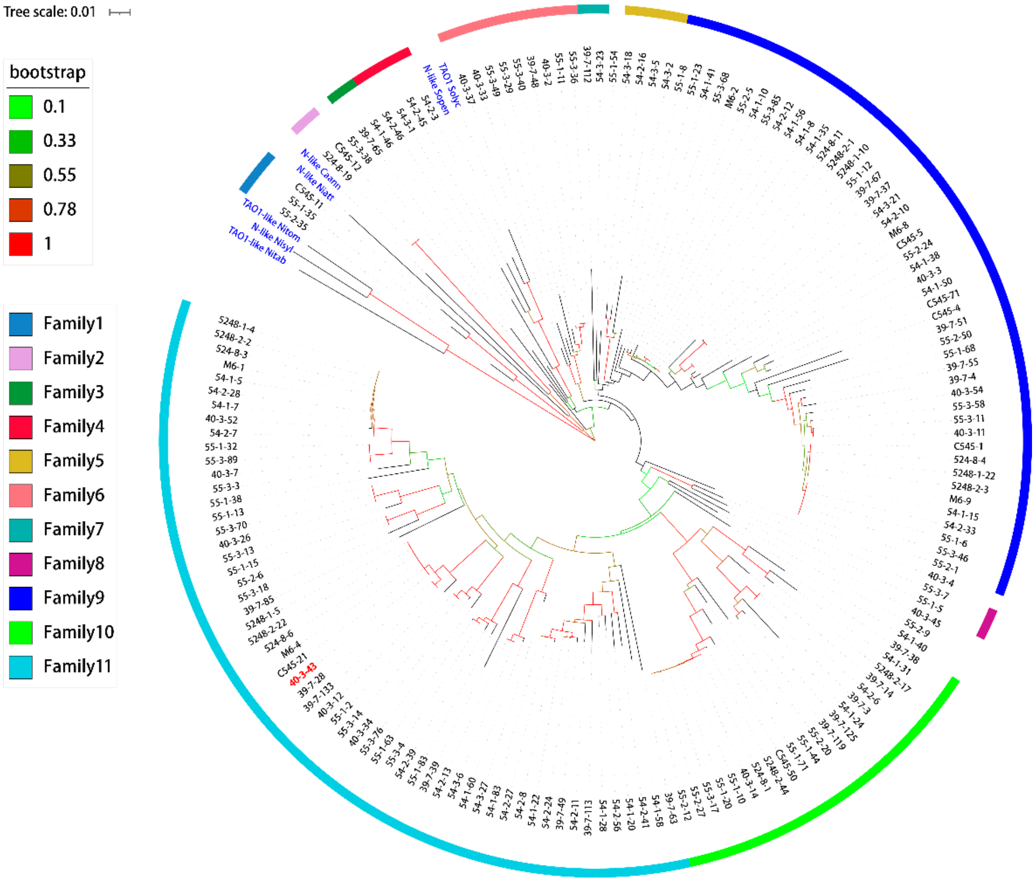


**Figure S5.** Phylogeny of *Ry*$_{chc}$ homologs. A neighbor-joining (NJ) phylogenetic tree was constructed for 13 diverse wild accessions of *S. chacoense*. Seven *Ry*$_{chc}$ homologs from other species (indicated with blue) were used as outgroups. These seven homologs are *N-like_Caann* from pepper, *TAO1_Solyc* and *N-like_Sopen* from tomato, and *N-like_Niatt*, *N-like_Nisyl*, *TAO1-like_Nitab* and *TAO1-like_Nitom* from tobacco. The fragment of *Ry*$_{chc}$ is indicated with red.

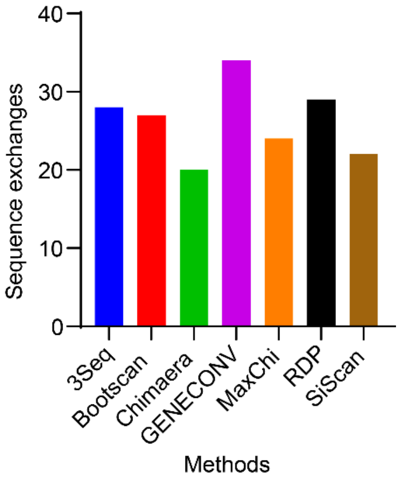


**Figure S6.** Sequence exchange in Family 11.

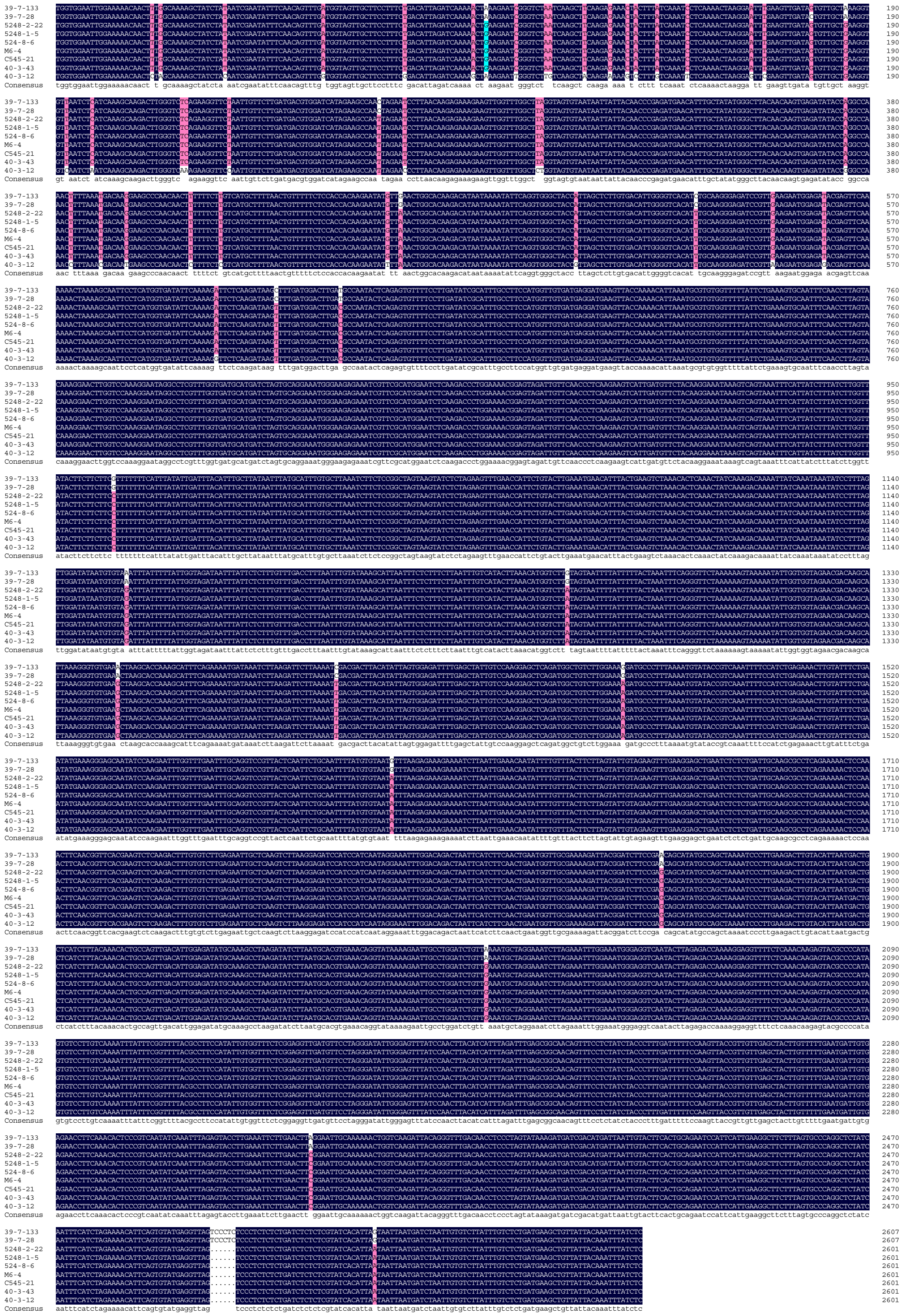

**Figure S7.** Genomic sequence alignment of the tight clade including 40-3-43.

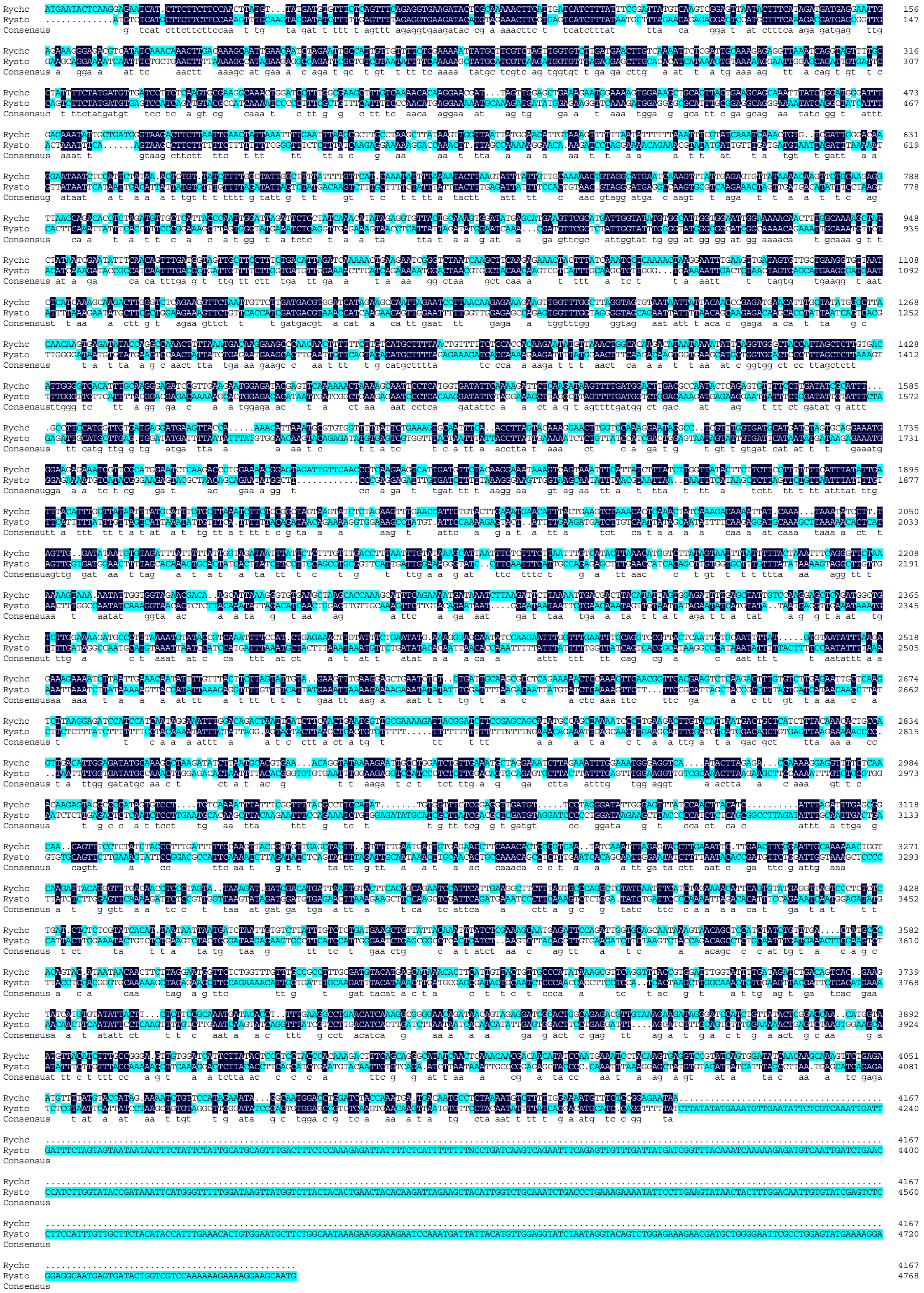

**Figure S8.** Genomic sequence alignment of *Ry*$_{chc}$ and *Ry*$_{sto}$.
